# Repeatability of Quantitative Magnetic Resonance Imaging Biomarkers in the Tibia Bone Marrow of a Murine Myelofibrosis Model

**DOI:** 10.3390/tomography9020045

**Published:** 2023-02-28

**Authors:** Brian D. Ross, Dariya Malyarenko, Kevin Heist, Ghoncheh Amouzandeh, Youngsoon Jang, Christopher A. Bonham, Cyrus Amirfazli, Gary D. Luker, Thomas L. Chenevert

**Affiliations:** 1 Department of Radiology and the Center for Molecular Imaging, University of Michigan School of Medicine, Ann Arbor, MI 48109, USA; 2 Department of Biological Chemistry, University of Michigan School of Medicine, Ann Arbor, MI 48109, USA; 3 Department of Microbiology and Immunology, University of Michigan School of Medicine, Ann Arbor, MI 48109, USA

**Keywords:** quantitative MRI, repeatability, murine model of myelofibrosis, MF, bone marrow, BM, quantitative imaging biomarker, QIB, apparent diffusion coefficient, ADC, proton density fat fraction, PDFF, magnetization transfer ratio, MTR

## Abstract

Quantitative MRI biomarkers are sought to replace painful and invasive sequential bone-marrow biopsies routinely used for myelofibrosis (MF) cancer monitoring and treatment assessment. Repeatability of MRI-based quantitative imaging biomarker (QIB) measurements was investigated for apparent diffusion coefficient (ADC), proton density fat fraction (PDFF), and magnetization transfer ratio (MTR) in a JAK2 V617F hematopoietic transplant model of MF. Repeatability coefficients (RCs) were determined for three defined tibia bone-marrow sections (2–9 mm; 10–12 mm; and 12.5–13.5 mm from the knee joint) across 15 diseased mice from 20–37 test-retest pairs. Scans were performed on consecutive days every two weeks for a period of 10 weeks starting 3–4 weeks after transplant. The mean RC with (95% confidence interval (CI)) for these sections, respectively, were for ADC: 0.037 (0.031, 0.050), 0.087 (0.069, 0.116), and 0.030 (0.022, 0.044) μm^2^/ms; for PDFF: 1.6 (1.3, 2.0), 15.5 (12.5, 20.2), and 25.5 (12.0, 33.0)%; and for MTR: 0.16 (0.14, 0.19), 0.11 (0.09, 0.15), and 0.09 (0.08, 0.15). Change-trend analysis of these QIBs identified a dynamic section within the mid-tibial bone marrow in which confident changes (exceeding RC) could be observed after a four-week interval between scans across all measured MRI-based QIBs. Our results demonstrate the capability to derive quantitative imaging metrics from mouse tibia bone marrow for monitoring significant longitudinal MF changes.

## 1. Introduction

Myelofibrosis is a chronic, ultimately fatal myeloproliferative neoplasm (MPN) characterized by progressive fibrosis of bone marrow, dramatic elevations or reductions in blood cells, profound hepatosplenomegaly, and debilitating constitutional symptoms [1,2,3]. Almost all cases of MF arise from genetic mutations in one of three key genes in hematopoietic stem or progenitor cells (HSC) [4]. Up to 20% of patients progress to acute myelogenous leukemia (AML), a complication that accelerates fatal progression of disease [5]. Currently, biopsy remains the only method to assess bone marrow (BM), the primary site of disease in MF [6]. Bone-marrow biopsies inherently suffer from sampling error, as the technique only analyzes millimeter amounts of tissue from one anatomic site (iliac crest). Biopsy also cannot assess anatomic heterogeneity of disease [7], a common feature of MF based on autopsies. In patients with extensive fibrosis, BM biopsies frequently recover no tissue (“dry tap”), leaving physicians with no information about bone-marrow composition and severity of disease.

As a participant of the NIH/NCI Co-Clinical Imaging Research Resource Program (CIRP) [8] our team seeks to develop and validate MRI methods to quantify bone-marrow composition and architecture in co-clinical trials by evaluating image-based biomarkers for monitoring disease trajectory and response to treatment. In the pre-clinical arm of this co-clinical trial, serial MRI studies aim to assess the ability of quantitative imaging readouts to accurately detect changes in underlying bone marrow phenotypes and assess disease trajectory in mouse models of myelofibrosis [9]. Driven by the major mutation causing MF in patients, the JAK2 V617F hematopoietic transplant mouse model recapitulates human MF bone marrow phenotypes which are the target of MR scanning protocols to detect fibrosis, altered fat content, and hypercellularity during MF disease progression, with concomitant increase in spleen volume (splenomegaly) due to extramedullary hematopoiesis [9]. These disease-induced phenotypes are being assessed using three MRI techniques including diffusion-weighted imaging (DWI) to derive apparent diffusion coefficient (ADC) reflecting water mobility sensitive to cellular density [10,11]; chemical shift imaging to derive proton density fat fraction (PDFF) [12,13]; and magnetization transfer ratio (MTR) for monitoring water binding affinity changes in extracellular macromolecules (i.e., in fibrosis) [14,15,16]. In parallel with our human MF imaging, changes in mouse spleen volumes [17] are also assessed over time using 3D anatomical MRI, although this is outside the focus of this work.

An ideal imaging biomarker of disease would be a constituent that reflects a related phenotype, and that can undergo measurable change in response to perturbation. Often biomarkers represent biological or biophysical targets of disease that can be clinically monitored for diagnostic or treatment response [18,19,20]. Measurement precision, or repeatability, relative to disease-/treatment-induced changes is critical for effective disease monitoring and/or determination of efficacious response. Evaluation of murine tibia bone marrow (which occupies a space <1 mm in diameter) at sufficient sensitivity and spatial resolution to allow reliable quantification of changes over time using image-based biomarkers represents additional challenges. To overcome these challenges, imaging the immobilized murine extremity within the confined cryogenic coil limits motion artifacts and provides adequate high signal-to-noise ratio (SNR) and resolution for sampling spatial disease nonuniformity along tibia BM.

Here, we investigated the measurement precision of ADC, PDFF, and MTR quantitative imaging biomarkers (QIBs) in murine tibia bone marrow using a test (TT)–retest (RT) experimental design wherein tibia BM imaging was performed on consecutive days under the assumption that biological variation remained constant as compared to more progressive changes occurring over several weeks/months typical of MF disease evolution. Differences in the measured biomarker TT and RT values were used to calculate precision metrics such as the repeatability coefficient (RC) [21,22], where multiple TT–RT pairs acquired over several weeks/months after transplantation may establish precision estimates that reliably represent diseased mice. In the context of human trials [13,22,23,24], confident detection of a significant change in an individual requires the biomarker value change to exceed measurement uncertainty (i.e., RC). To parallel human application, detection of significant change was assessed by analysis of longitudinal differences between baseline measurements among individual mice relative to RC to identify differences reflected by these imaging biomarkers relative to their precision.

## 2. Materials and Methods

Repeatability coefficients (RCs) for ADC, PDFF, and MTR imaging biomarkers in murine model of myelofibrosis (MF) disease were determined from test–retest imaging studies. The imaging schedule consisted of paired test (TT)–retest (RT) scans being acquired on consecutive days for each animal starting ~28 days post bone-marrow transplant (BMT). Sequential TT–RT pairs among individual animals were spaced approximately 10 to 14 days apart, with a total of 37 scan pairs collected across 15 diseased mice over several months after transplantation (1–5 TT–RT pairs per mouse), providing a large data set for evaluation of quantitative MRI biomarker repeatability. The TT–RT sessions were completed for all animals during their scheduled scan series prior to image-quality review. The six wild-type (WT) non-diseased mice that did not undergo irradiation or BMT were imaged for a total of 27 scans over one month. The application of RC-based analysis was demonstrated for initial changes between non-ablated wild-type mice without disease and ablated diseased mice post bone-marrow transplant, as well as for characterization of spatiotemporal trends in imaging biomarkers among the diseased mice.

### 2.1. Animal Model of Myelofibrosis

All animal procedures were approved by the University of Michigan Institutional Animal Care and Use Committee. Rodents were maintained in a specific pathogen-free barrier unit at the University of Michigan accredited by the Association for Assessment and Accreditation of Laboratory Animal Care. Euthanasia of mice at the end of the study followed guidelines described for use with end-stage illness and humane endpoints. Wild-type female C57Bl/6 mice were purchased from Charles River Laboratories (Wilmington, MA, USA).

The JAK2 V617F (Jak2^+/VF^) animal model of myelofibrosis (MF) was generated using resultant 8–10-week-old female donor offspring from a cross between Jak2^+/Fl^ mice (B6N.129S6(SJL)-Jak2^tm1.2Ble^/AmlyJ; Jackson Laboratory Stock No. 031658) and Mx-Cre mice (B6.Cg-Tg(Mx1-cre)1Cgn/J; Jackson Laboratory Stock No. 003556), similar to previously described methods [9,20]. Briefly, whole bone-marrow cells were isolated from donor mice and mixed 1:1 with whole bone-marrow cells isolated from age- and gender-matched wild-type C57Bl/6 mice. A total of 1 × 10^7^ mixed bone-marrow cells was injected retro-orbitally into lethally irradiated 6-week-old female recipient C57Bl/6 mice. Polyinosinic–polycytidylic acid (10 mg/kg) was administered intra-peritoneally 10 days post bone-marrow transplant (post-BMT) for induction of Cre recombinase-mediated replacement of the floxed endogenous exon with the mutated exon of Jak2 for expression of the JAK2 V617F mutant allele. Six age- and gender-matched wild-type C57Bl/6 mice did not undergo irradiation, bone-marrow transplant, or disease induction.

For MRI studies, animals were anesthetized using a 1.5% isofluorane/air inhalation mixture, monitored for respiratory sufficiency using an Small Animal Instruments monitor (SAI Inc., Stony Brook, NY, USA), and thermoregulated using a 37 °C heating bed to maintain body temperature during imaging. Anesthetized mice were positioned in a holder allowing for the leg to be held in position by a 3D-printed, leg-shaped mold on the posterior side and a CryoProbe^TM^ on the anterior side. Following imaging, animals were situated in an isolated cage until full recovery, then moved back to a communal cage.

### 2.2. MR Imaging Scanner Hardware

All MR scans were acquired using a Bruker BioSpec^®^ MRI Console (Bruker Preclinical MRI, Billerica, MA, USA) with Paravision 7.0.0 software installed on 64-bit Linux multicore workstation attached to a 7 Tesla, 30 cm horizontal bore magnet model “7T/310/AS” System (Agilent) (300 MHz ^1^H frequency). The system has a gradient/shim coil set with inner diameter 114 mm, gradient strength 440 mT/m, max slew rate 3440 T/m/s, 10 shim channels, and up to 4th order shim coils. A large transmit/receive RF volume coil with outer/inner diameter 112 mm/86 mm was used in tandem with a small receive CryoProbe^TM^ 4 element array RF coil which was cryogenically cooled to 20–30 K to enhance signal-to-noise ratio (SNR) as required to image bone marrow of murine tibia at high sensitivity and spatial resolution [25,26]. Standard Bruker on-scanner reconstruction algorithms were used to generate all MR images at acquired contrasts for off-line conversion to biomarker maps described below.

### 2.3. DWI Acquisition Technique

A single spin-echo, 3-orthogonal diffusion weighted axes, one phase-encode per repetition time (TR), i.e., non-echo-planar imaging, protocol was used to acquire diffusion weighted imaging (DWI) scans using a 2D multi-slice sequence in the coronal plane (see Appendix A for acquisition geometry). Acquisition parameters were: TR/TE = 2000/22 ms; spectral fat-suppression; 2 number-of signal-averages (NSA); established diffusion nomenclature [11], b-values = 0 and 3000 s/mm^2^ on each (*X*, *Y*, *Z*) axis; diffusion pulse timing δ = 4 ms, Δ = 10 ms; and total scan time = 17 min. Conventional 2D Cartesian full k-space coverage was performed. Physiologic synchronization was not required. Bruker standard reconstruction algorithms were used to generate directional DWI sets with nominal b = 0 and 3000 s/mm^2^ weighting.

### 2.4. ADC Biomarker Map Generation

Image files in Bruker-native format were converted to apparent diffusion coefficient (ADC) maps offline using custom routines in Matlab version R2019b (MathWorks Inc., Natick, MA, USA) [27]. Briefly, pixels having less than 20% of mean signal at *b* = 3000 s/mm^2^ or having lower signal at *b* = 0 compared to *b* = 3000 s/mm^2^ were excluded. Following standard procedures, directional *ADC_j_* maps (*j = X*,*Y*,*Z*) were calculated using actual directional *b*-values (*b_j_*) gleaned from associated Bruker text files, then averaged over three directions to generate an isotropic ADC map, and an isotropic *DWI_trace_* was calculated from the geometric mean of the directional DWI:

(1) $${ADC}_{j}=\frac{1}{b_{j}}ln\left[\frac{{DWI}_{b0}}{{DWI}_{b_{j}}}\right];ADC=\left[{ADC}_{X}+{ADC}_{Y}+{ADC}_{Z}\right]/3$$

(2) $${DWI}_{trace}=\sqrt[{\frac{1}{3}}]{\left[{DWI}_{b_{X}}\cdot {DWI}_{b_{Y}}\cdot {DWI}_{b_{Z}}\right]}$$

### 2.5. PDFF Acquisition Technique

Multi-echo chemical shift encoding was used to decompose water and fat signal constituents at 7 Tesla requiring echo-to-echo spacing of approximately 0.4 ms or less, which is difficult to achieve in a single echo train while maintaining high spatial resolution to image mouse tibia bone marrow. Instead, four consecutive series were acquired for retrospective combination, where each was a 3-echo, gradient-echo series. Echo spacing between the three echoes was held constant at 2.174 ms, although echo time (TE) of the first echo was incremented over the four consecutive series as follows: Series1 = 1.476 ms; Series2 = 1.793 ms; Series3 = 2.110 ms; and Series4 = 2.427 ms. Hardware settings, acquisition geometry, shim, transmit gain, and receiver gains were all held constant over the four consecutive series such that the four series data could be retrospectively combined and sorted by TE into an effective 12-echo train. Assuming the spectrometer was stable throughout acquisitions, this scenario samples the evolution between water and fat signal constituents with 0.317 ms temporal resolution, which is adequate for chemical shift signal decomposition at 7 Tesla. Complex-valued 3D image sets were reconstructed for each of the 12-echo times using standard on-scanner Bruker image reconstruction routines. See Appendix A for acquisition geometry of the 3D multi-echo, gradient echo (MGE) sequence. Other key acquisition parameters included: TR = 50 ms; flip angle = 5°; 2 NSA; total scan time for the four MGE series = 4 × 13.7 min = 55 min.

### 2.6. PDFF Biomarker Map Generation

Constituent water and fat contributors to MGE signals were decomposed using custom offline Matlab scripts based on a seven-peak water–lipid model and graph-cut algorithm [28]. Complex-valued 3D images set at each of 12-echo times, stored in Bruker-native format, were input into the water–fat decomposition script along with a list of echo times, proton frequency and geometry parameters read from Bruker text files. Fat–water decomposition depends on graph-cut parameters, image SNR, and magnetic field (B0) homogeneity adjusted during each scan session. Matlab script output images of water (W), fat (F), PDFF defined as 100% F/(W+F), and mean MGE signal over 12 echoes.

### 2.7. MTR Acquisition Technique

Magnetization transfer contrast was induced using the standard Bruker 3D fast low-angle shot (FLASH) sequence in two consecutive 3D FLASH series wherein the first was without an additional off-resonance RF saturation pulse (MToff), and the second was with an additional Gaussian-shaped, 8 µT amplitude, 100 ms duration RF saturation pulse at negative –2400 Hz off-resonance (MTon). Hardware settings, acquisition geometry, shim, transmit gain, and receiver gains were held constant over the two consecutive series such that measurable change in signal between MToff and MTon was presumed related to magnetization transfer modulated by macromolecules in the solid tissue matrix (including fibrosis). Acquisition geometry of the 3D FLASH series is provided in Appendix A. Other key acquisition parameters included: single-echo; TR/TE = 111 ms/2.99 ms; flash-spoiling, flip angle = 9°; 1 NSA; total scan time = 15 min × 2 = 30 min for both series.

### 2.8. MTR Biomarker Map Generation

Time-domain 3D FLASH data were converted into magnitude-valued space-domain MToff and MTon images using standard on-scanner Bruker reconstruction routines and stored in Bruker-native image file format. Offline custom Matlab scripts were used to generate 3D MTR maps, defined as MTR = (MToff – MTon)/MToff [14,15,16]. The maps were calculated on pixel-by-pixel basis for sufficient SNR (>20) pixels. Pixels below this threshold were set to zero in MTR maps and excluded from analysis.

### 2.9. Tibia Bone Marrow Segmentation

**Figure 1:**
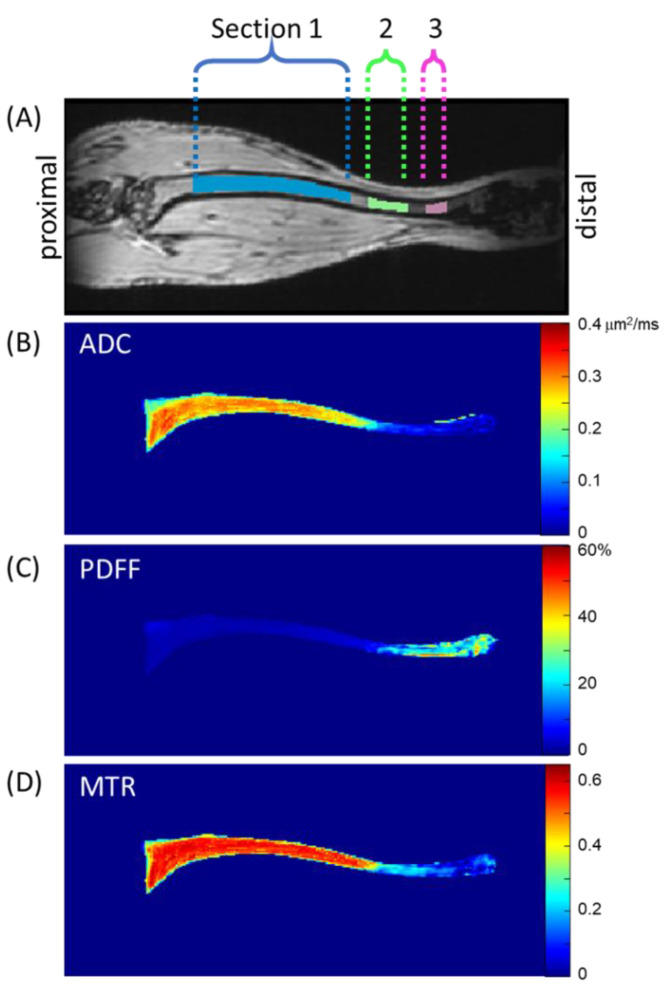
(**A**) Coronal slice through volumes of interest (VOI) along length of tibia shows Sections S1–S3. For the image shown, volumes in S1, S2, and S3 were 3.5, 0.3, and 0.2 mm^3^, respectively. Coronal projections of mean biomarker value within the tibia bone marrow are shown for biomarker maps (**B**) ADC in µm^2^/ms units, (**C**) PDFF in %, and (**D**) MTR (dimensionless).

Matlab scripts generating ADC, PDFF, and MTR maps along with select source images were output in 3D Meta image header (MHD) format [29] for convenient input into publicly available image analysis/segmentation platforms such as 3DSlicer [30]. Murine tibia images were manually segmented in coronal sections by an experienced (>3 years) image analyst (K.H.) on 3D MToff images and stored as a binary volume of interest (VOI) mask in MHD format. Inter-observer repeatability was not tested in this study. Inspection of diseased mouse tibia bone marrow after transplantation by MRI revealed contrast heterogeneity primarily as a function location along the length of the tibia; therefore, three sections were automatically parsed from each stored VOI mask, as follows (see Figure 1). The axial slice containing the largest count of pixels within the VOI mask was assigned z-location 0 mm near the proximal end of the tibia. Pixels within the VOI mask spanning z-location 1.8 to 9 mm were defined as Section 1 (S1). Similarly, the span between 9.8 and 11.7 mm was defined as Section 2 (S2), and between 12.6 and 13.5 mm as Section 3 (S3).

Figure 1 illustrates these sections through one coronal MToff image, although the tibia bone marrow extended through several coronal slices. To illustrate representative biomarker map features, mean values projected through coronal slices within the VOI mask are displayed on quantitative color scales in Figure 1B–D for ADC, PDFF, and MTR, respectively.

For a given MRI session, acquisition geometry was spatially linked across DWI, MGE, and FLASH acquisitions so that the VOI and derived sections S1, S2, and S3 were directly applicable to ADC, PDFF, and MTR maps for independent analyses within each section for all three biomarkers. Prior to analyses, data quality checks were performed to remove artefactual datasets. Mean section ADC values below 0.05 µm^2^/ms and mean section MTR values below 0.1 were automatically eliminated from ADC and MTR analyses, respectively. This resulted in the following exclusions of TT–RT pairs for ADC S1: 4/37, S2: 8/37, and S3:17/37; and for MTR S2: 9/37 and S3:15/37. This elimination most frequently occurred in S3 due to lower SNR at the fringe of the receiver coil and suppression of the more dominant fat signal (for DWI) in the distal end of the tibia bone marrow. PDFF and B0 homogeneity maps were visually screened by investigators (GA and TLC) to identify datasets with artefactual zones exhibiting apparent water–fat decomposition errors which were eliminated from further analysis (PDFF S2: 10/37 and S3: 15/37). These eliminations predominantly occurred in S3 due to relatively poor B0 homogeneity at the distal end.

### 2.10. Repeatability Analysis

Bland–Altman (BA) analysis was used to evaluate test (TT)–retest (RT) repeatability of the quantitative imaging biomarkers (QIBs). For scan pairs acquired on consecutive days, QIB values are not expected to change significantly, thus the differences in the measured QIBs should ideally be zero. The repeatability and comparability of QIBs estimated from the TT–RT pairs were assessed using the within-subject standard deviation (wSD) and BA analysis for limits of agreement (LOA) [22,31]. wSD was calculated as an average standard deviation of the section-specific mean QIB for the TT–RT scan pairs with confidence intervals (CIs) defined as

(3) $$95\%CI(wSD)=wSD\cdot \left[\frac{1}{\sqrt{\left(N-1\right)\cdot \chi_{0.975}^{2}}};\frac{1}{\sqrt{\left(N-1\right)\cdot \chi_{0.025}^{2}}}\right]$$

The repeatability coefficient (RC) was further calculated as RC = 2.77⋅wSD (in QIB units) [22,31]. Note, high RC values indicate high variability. The BA bias (average (QIB_RT_–QIB_TT_)) and limits of agreement (LOA=bias ± 1.96∙SD(QIB_RT_-QIB_TT_)) together determine the similarity between the TT-RT QIB pairs. Small bias and narrow LOA indicate that the two measurements are essentially equivalent.

For repeatability analysis, test and retest scans of 15 diseased mice were analyzed over the period of ~3–4 to 13–14 weeks after ablation and transplantation. The section-specific mean values of TT–RT QIB pairs were calculated for individual tibia bone-marrow sections (Figure 1) and used when measured values for both TT and RT scans were available following stated data quality screening procedures. Since data quality screening was performed independently for each section, the quantity of usable TT–RT pairs varied across sections (20–37 pairs). For the non-ablated wild-type (WT) mice without disease, only three had repeated scans (total of six pairs), not sufficient for RC analysis (hence, not performed) [31]. Five of the WT mice had 2–5 longitudinal scans (starting at 7–8-weeks post-ablation of the diseased mice), respectively. These were averaged to establish section-specific QIB values for each individual animal assuming no significant time-dependence over the course of the study due to lack of disease in WT mice. This assumption was supported by time-dependent linear regression analysis, *p*-values > 0.14.

### 2.11. QIB Longitudinal Trend Analysis

By comparison to derived RC thresholds, significant changes in QIB values for individual animals over time can be detected with 95% confidence [21,22]. To illustrate this application, QIB change-trend analysis was performed for 13 animals that had longitudinal imaging series ranging from 2–5 scans, taken every ~2 weeks over the study duration, starting ~3–4 weeks post-ablation. Specified QIB changes in individual bone-marrow sections for each animal were determined from the difference between a defined timepoint to a reference point, compared to the section-specific RC threshold.

For analysis between diseased and wild-type mice, the reference point was the collective section-specific average among all six WT animals. For analysis of diseased mice over time, the reference point was the section-specific QIB mean value (above) from each animal’s baseline (BL) scan (5–6 weeks post-ablation for ADC; 3–4 weeks post-ablation for PDFF and MTR), as diseased mice were not imaged prior to disease induction. The QIB changes exceeding RC thresholds were considered significant with 95% confidence [21,22], and the number of animals that displayed significant changes for individual section-specific QIBs was recorded for each imaging point.

## 3. Results

**Figure 2:**
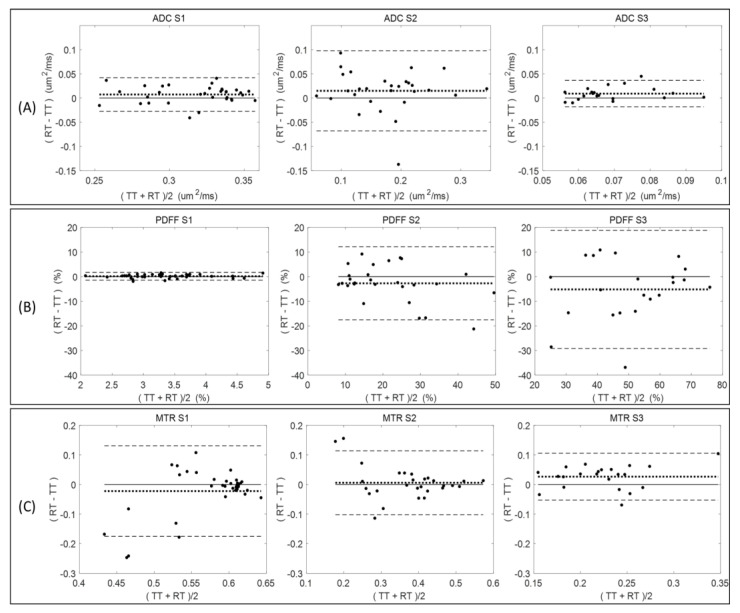
Bland–Altman plots graphically illustrate biomarker repeatability between paired test (TT) and retest (RT) measurements for (**A**) ADC; (**B**) PDFF; and (**C**) MTR. Plots for sections S1, S2, and S3 are in the left, center, and right columns, respectively. Mean difference (bias) is shown as dotted lines, and dashed lines represent limits of agreement (LOA). Vertical scale is held constant for each given biomarker to illustrate section-dependent repeatability (i.e., location along tibia—see Figure 1). Refer to Table 1 for number of TT–RT pairs in each plot.

Figure 2 summarizes the Bland–Altman analysis for paired test (TT)–retest (RT) scans of diseased mice across all measured QIB metrics (ADC, PDFF, and MTR) within the defined tibia bone-marrow sections (S1, S2, and S3). The total number of available TT–RT pairs decreased for S3 among all QIBs due to generally observed lower measurement quality (Table 1). Comparing tibia bone-marrow sections S1 to S3, the ADC and MTR ranges (Figure 2, horizontal axis scale) are decreasing, and PDFF ranges are increasing. The minor bias (mean difference) in ADC is positive for all sections, while negative for S2 and S3 in PDFF, and changing from negative in S1 to positive in S2 and S3 for MTR (Figure 2 and Table 1).

Negative bias for PDFF in S2 and S3 indicate the tendency for lower PDFF values in retest measurements, although the negative bias was small relative to the LOA. The LOA increased for PDFF from S1 (highest agreement) to S3 (least agreement), while decreasing for MTR (least agreement for S1 and highest for S3). The ADC repeatability (LOA) were comparable for S1 and S3, but S2 LOA were about twice as broad (Figure 2).

Sufficient quantity of test–retest pairs from diseased mice (20–37 pairs) was available to allow for RC determination with reasonable confidence intervals (Table 1). Repeatability values were spatially dependent among individual tibia bone-marrow sections. For example, PDFF measures had high RC values (indicating large variability) and a notable negative bias (consistent with Figure 2) between test and retest scans for S2 and S3 as compared to S1 (smaller RC and bias; Table 1). The difference between mean QIB values for S1, S2, and S3 exceeded the highest RC among individual sections, confirming substantial spatial heterogeneity of bone marrow along the tibia, and supporting our decision for independent analysis of each section (Table 1).

**Figure 3:**
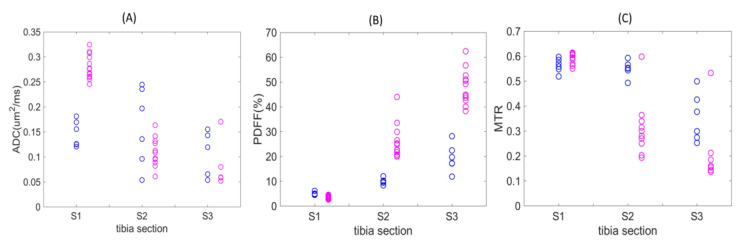
QIB values at 5–6 weeks post-ablation for individual diseased mice (magenta) and wild-type (non-ablated) mice (blue, average of 2–5 longitudinal measurements) across the defined tibia bone-marrow sections. Plots for ADC (**A**), PDFF (**B**), and MTR (**C**).

Figure 3 displays QIB values for individual diseased mice at weeks 5–6 post-ablation (magenta) and longitudinal average QIB values for non-ablated wild-type C57Bl/6 mice (blue) across each of the three defined tibia bone-marrow sections. For ADC values, complete separation was observed for S1, with the diseased mice ranging from ~0.25 to 0.33 μm^2^/ms as compared to non-ablated wild-type mice (<0.20 μm^2^/ms; Figure 3A). While PDFF values from S1 were similar for diseased and the wild-type animals, sections S2 and S3 both revealed higher PDFF values in diseased mice (Figure 3B).

However, MTR measures revealed mostly lower values for diseased bone marrow in Sections S2 and S3 as compared to WT mice (Figure 3C). Section S2 and S3 ADC values for WT mice displayed high variability, and only a smaller number of confident measurements was possible for diseased mice in S3 (5 of 13; Table 2). For PDFF and MTR of wild-type mice, the QIB gradient is evident in the spatial transition from sections S1 to S3 (Figure 3B,C, Table 2).

With respect to non-ablated WT mice, diseased mice at weeks 5–6 post-ablation showed significant change (majority exceeding RC, Table 1) in ADC among all sections, and among S2 and S3 in PDFF (increasing) and MTR (decreasing), across a large fraction (0.75–1.0) of animals (Table 2).

**Figure 4:**
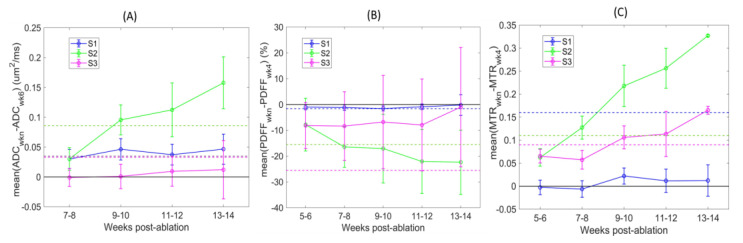
QIB changes with respect to initial BL imaging points (post-ablation week 4 for MTR and PDFF, and week 6 for ADC) for individual animals are plotted for ADC (**A**), PDFF (**B**), and MTR (**C**). Symbols and error bars mark, respectively, the average QIB change and its standard deviation across animals. The tibia bone-marrow section assignments are color-coded in the legend. The solid lines connect symbols to illustrate the trends, and the dashed horizontal lines mark the 95% confidence threshold for the corresponding QIB changes (determined by RC in Table 1).

The spatial gradient of section-mean PDFF and MTR values, respectively, increasing and decreasing towards more distal sections of the tibia (from S1 to S3), was consistently observed over time (Table 3). The QIB heterogeneity (SD) across animals was the highest for S3 PDFF and S2ADC, and slightly increased with time. The number of diseased animals with confident S3 measurements at initial baseline (BL) imaging was low (Table 3) for ADC (5/13) and MTR (4/13), apparently reflecting limited sensitivity of DWI and MT acquisition protocols in this (small) tibia bone-marrow section with high fat content at the distal edge of the imaged volume (Table 3). For PDFF in S1, the fat fraction was relatively constant with respect to time. The most notable changes over time were observed for mid-tibia section S2 across all QIB metrics; section-mean values gradually increased for ADC and MTR, and decreased for PDFF. Notably, the number of mice with significant changes (>RC) in S2 gradually increased over time, with significant changes also being observed for most mice at later timepoints for ADC in S1 and MTR in S3 (Table 3 and Figure 4).

The QIB differences over time relative to initial baseline (BL) reference imaging points are further illustrated in Figure 4. Detected changes varied depending on the defined tibia bone-marrow sections (color-coded). The changes were mostly positive for ADC and MTR, and negative for PDFF post-ablation. These changes are opposite to those observed between week 5 and 6 post-ablation for non-ablated wild-type animals (Table 2), likely indicative of a response to restore the bone-marrow niche after ablation and transplantation. The largest differences over time were observed for mid-tibia S2 (green) across all QIBs, and 4 weeks from initial BL imaging points (7–10 weeks post-ablation). These changes exceeded RC thresholds (dashed lines) for most animals (Figure 4 and Table 3), indicating that the changes were detectible with the 95% confidence. Less prominent changes in ADC of S1 and MTR of S3 were also confidently detected for most animals after 6 weeks from initial imaging scans (9–10 weeks post-ablation).

## 4. Discussion

Our study was designed to determine MRI-derived repeatability measures of ADC, PDFF, and MTR quantitative biomarkers (QIBs) from tibial bone marrow in a mouse model of myelofibrosis (MF). Test–retest MRI scans for measured QIBs established repeatability coefficients with confidence intervals that were applied to longitudinal monitoring for detection of significant changes over time. This study revealed substantial heterogeneity of ADC, PDFF, and MTR values within the tibia bone marrow that we defined by three distinct sections along the bone axis (proximal to distal) and showed to have tibia-segment dependent repeatability coefficients (RCs). Overall, the MTR and PDFF variability (RC) increased with increasing QIB value, while ADC variability was the highest for intermediate ADC values; however, each bone-marrow section exhibited differential evolution in underlying QIBs over time.

Both ADC and MTR of the bone marrow decreased toward the distal tibia but generally increased with time, while PDFF increased along the tibia and generally decreased with time. Thus, the use of noninvasive MRI enabled monitoring of spatiotemporal changes within mouse tibia bone marrow (BM) following ablation and transplantation. Furthermore, based on the QIB change-trend analysis, we report that an imaging schedule of every 4 weeks is sufficient to detect confident changes (exceeding RC) in tibia bone marrow of this murine MF model with the currently described QIB MRI protocols. Although larger repeatability errors were observed for PDFF, MRI was determined to provide sufficient sensitivity to detect progressive changes over time in mice, suggesting that additional imaging protocol improvements may assist to further reduce PDFF errors, thereby increasing detection sensitivity. Overall, imaging protocol improvements (e.g., higher SNR and reduced artifact) toward enhanced repeatability versus reported baseline performance would enable higher sensitivity for early detection of disease-induced changes and for progression trend analysis of individual animals.

The observed PDFF changes were consistent with post-ablation BM repopulation in prior studies of age- and gender-matched C57Bl/6 mice transplanted with normal marrow [32]. This study observed a clear proximal to distal gradient of histologic adiposity prior to ablation, particularly in the murine femur bone marrow [32]. Upon ablation and transplantation, restoration to initial adiposity levels occurred over approximately six weeks post-ablation, displaying a clear proximal to distal kinetic adiposity gradient in the murine femur [32]. A similar trend over time from high to low PDFF post-ablation was also observed in the mid-tibia bone-marrow section in our study indicating the potential for PDFF as a suitable QIB for monitoring temporal evolution of marrow. In addition to PDFF, detectable changes with respect to wild-type mice were observed for ADC and MTR at the first imaging point of diseased mice in different tibia sections. These observations hold promise for earlier detection and suggest complementary QIB sensitivity to different pathology manifestations (e.g., ADC for inflammation and MTR for fibrosis). Moreover, finer sub-region analysis including voxel-wise QIB differences may improve sensitivity to early change compared to mean change over large tibia sections, as described here.

The main limitations of the present study were in using a single MRI hardware with an optimized imaging protocol for a specific site of disease. Therefore, reported repeatability values are relevant for the studied disease site (tibia BM) and high SNR cryogenic coil imaging and cannot be assumed for other organs or acquisition protocols. Manual tibia segmentations could also have increased the observed repeatability errors. However, providing the baseline RC values is a prerequisite for setting a benchmark for protocol improvement and is an illustration of necessary steps for confident analysis of time-trends for progression in an individual animal. The translation of corresponding QIB to human MF studies should follow similar workflow for whole body imaging protocols.

Overall, these MRI studies establish and provide foundational methodologies necessary for conducting multidimensional multiparametric image acquisition, digital image processing, and statistical analysis of tibia BM scans from mouse models of myeloproliferative neoplasms (MPNs). This approach can be used to both facilitate quantitative evaluation of clinically approved therapeutic interventions and compare these results to novel experimental drugs [18,19]. Additionally, the application of quantitative DWI, which has been broadly used for assessing rodent tumor model treatment responses [19,33,34], can be extended along with additional metrics (i.e., MTR and PDFF) to assess MPN disease models in a highly rigorous and noninvasive longitudinal monitoring of disease. Multimodal MRI evaluation of bone-marrow cancers is also envisioned to be used to assess the biological impact of various genomic mutations on both tissue phenotypes during disease progression and their relative responses to treatment intervention. Thus, quantitative noninvasive MR imaging biomarkers can facilitate understanding of the interplay between the underlying genomic landscape(s) of MF and disease pathophysiology within the bone marrow.

Quantitative MRI is positioned to provide a major advance in tools available to design novel therapeutic strategies and the outcomes of patients with MPNs. As each of the measurements presented herein is clinically translatable, these approaches provide a unique opportunity to develop companion diagnostic imaging biomarkers for disease staging and monitoring of novel therapeutic regimens in clinical trials. While the studies here were focused on myelofibrosis, the MRI methods can be extended to support imaging and data-analysis protocols for quantitative analysis of bone marrow in co-clinical trials of multiple other hematologic cancers. Our currently active clinical trial (NCT01973881), as part of the CIRP, continues to translate animal-derived methodologies to investigate quantitative BM MRI biomarkers as novel response metrics for patient assessment [35,36].

## 5. Conclusions

Our comparative analysis of MRI quantitative biomarkers in a mouse model of myelofibrosis provided an opportunity to define reproducibility metrics which were found to be spatially dependent within the tibia bone marrow. The presented approach can be implemented in future studies to improve longitudinal assessment of MPN disease phenotypes using MRI to provide both quantitative and precise spatial information. The ability to quantify disease-associated evolution of tissue changes and treatment response within the bone-marrow parenchyma offers an important resource to cancer researchers to improve and more rapidly evaluate therapeutic interventions urgently needed for patients with MPNs.

## Figures and Tables

**Table 1:** Repeatability statistics for diseased mouse tibia bone-marrow section QIBs.

QIB	Tibia Section	TT–RT Pairs	Mean	Bias	wSD	(wSD 95% CI)	RC	(RC 95% CI)
ADC (μm^2^/ms)	S1	33	0.317	0.008	0.013	(0.011, 0.018)	0.037	(0.031, 0.050)
	S2	29	0.175	0.014	0.031	(0.025, 0.042)	0.087	(0.069, 0.116)
	S3	20	0.069	0.009	0.011	(0.008, 0.016)	0.030	(0.022, 0.044)
PDFF (%)	S1	37	3.35	0.14	0.56	(0.46, 0.73)	1.6	(1.3, 2.0)
	S2	27	21.85	−3.51	5.58	(4.5, 7.3)	15.5	(12.5, 20.2)
	S3	22	50.31	−6.90	9.2	(7.5, 11.9)	25.5	(12.0, 33.0)
MTR	S1	37	0.58	−0.02	0.06	(0.05, 0.07)	0.16	(0.14, 0.19)
	S2	28	0.37	0.01	0.04	(0.03, 0.05)	0.11	(0.09, 0.15)
	S3	22	0.22	0.03	0.03	(0.03, 0.05)	0.09	(0.08, 0.15)

**Table 2:** Mean QIB values for WT mice and 5–6 weeks post-ablation for MF mice.

QIB	ADC (μm^2^/ms)			PDFF (%)			MTR		
Tibia section	S1	S2	S3	S1	S2	S3	S1	S2	S3
WT: mean (SD)	0.15 (0.03)	0.18 (0.06)	0.11 (0.05)	4.8 (0.2)	9.6 (0.7)	17.7 (3.9)	0.56 (0.03)	0.55 (0.04)	0.38 (0.09)
MF: mean (SD) #(>RC)/total	0.28 (0.02) * 13/13	0.11 (0.03) * 8/11	0.08 (0.05) * 3/5	3.5 (0.6) 1/12	26.0 (7.0) * 9/12	48.1 (7.1) * 10/12	0.56 (0.08) 0/11	0.31 (0.12) * 9/10	0.21 (0.13) * 7/8

“#(>RC/total)” lists the number of diseased animals with QIB changes exceeding RC with respect to the first available reference imaging point. * marks where more than half of diseased animals exceeded mean QIB of WT mice by the RC.

**Table 3:** Mean and SD QIB values for 13 diseased animals that had longitudinal imaging starting 3–4 weeks after bone marrow ablation.

	QIB	ADC (μm^2^/ms)			PDFF (%)			MTR		
Weeks post- ablation	Tibia section	S1	S2	S3	S1	S2	S3	S1	S2	S3
3–4	Mean (SD)	NA			4.4 (0.8)	34.2 (12.3)	53.8 (14)	0.57 (0.14)	0.22 (0.07)	0.12 (0.02)
	#(>RC)/ total	NA			BL			BL		
5–6	Mean (SD)	0.28 (0.02)	0.11 (0.03)	0.08 (0.05)	3.5 (0.6)	26 (7)	48.1 (7.1)	0.56 (0.08)	0.31 (0.12)	0.21 (0.13)
	#(>RC)/ total	BL			4/12	2/12	0/12	0/12	3/10	1/4
7–8	Mean (SD)	0.31 (0.02)	0.13 (0.04)	0.06 (0.02)	3.3 (0.6)	18.5 (9.4)	45.4 (16.9)	0.59 (0.03	0.33 (0.13)	0.17 (0.06)
	#(>RC)/ total	5/13	0/11	0/5	5/12	* 6/12	1/12	0/11	* 7/11	1/4
9–10	Mean (SD)	0.33 (0.01)	0.2 (0.03)	0.06 (0.01)	3 (0.5)	18.3 (8.5)	47.4 (16.3)	0.6 (0.04)	0.37 (0.12)	0.19 (0.07)
	#(>RC)/ total	* 10/13	* 8/11	0/5	* 6/11	* 6/11	2/11	0/10	* 9/10	* 2/4
11–12	Mean (SD)	0.32 (0.02)	0.21 (0.06)	0.07 (0.02)	3.7 (0.9)	13.3 (5.1)	46.3 (16.5)	0.6 (0.03)	0.44 (0.08)	0.21 (0.07)
	#(>RC)/ total	* 7/13	* 8/11	1/5	2/11	* 7/11	2/11	0/10	* 10/10	* 3/4
13–14	Mean (SD)	0.33 (0.01)	0.27 (0.04)	0.09 (0.01)	4.2 (3.9)	9.7 (6.2)	46 (23.3)	0.61 (0.01)	0.5 (0.1)	0.26 (0.08)
	#(>RC)/ total	* 5/7	* 6/7	* 3/5	3/7	* 5/7	2/7	0/6	* 6/6	* 4/4

NA: data not available since ADC b = 3000 s/mm^2^ measurement was not performed 3–4 weeks post-ablation. BL: reference baseline imaging point with respect to which the changes were assessed for individual animals/sections. “#(>RC/total)” lists the number of diseased animals with QIB changes exceeding RC with respect to the first available reference imaging point. *: denotes more than half of diseased animals exceeding their corresponding BL imaging QIB value by RC. The last timepoint had smaller number of animals (6–7, due to sacrifice) compared to earlier timepoints (10–13 animals). Smaller total numbers of mice for ADC and MTR section S3 are due to data quality issues at initial imaging points.

## Data Availability

Study data are available from the corresponding author on request.

## Appendix A

**Table A1:** DWI acquisition geometry.

Matrix	Acquired Voxel Size (μm)	FOV (mm)
128 (freq-enc on z-axis)	180 on z	23.04 on z
64 (phase-enc on x-axis	150 on x	9.6 on x
40 (slices, y-axis)	150 (slice thickness) on y	6.0 on y

**Table A2:** 3D MGE acquisition geometry.

Matrix	Acquired Voxel Size	FOV (μm)
256 (freq enc on z-axis)	90 on z	23.04 on z
128 (phase enc on x-axis	75 on x	9.6 on x
64 (phase enc on y-axis)	94 (slice thickness) on y	6.0 on y

**Table A3:** 3D FLASH acquisition geometry.

Matrix.	Acquired Voxel Size	FOV (μm)
256 (freq enc on z-axis)	90 on z	23.04 on z
128 (phase enc on x-axis	75 on x	9.6 on x
64 (phase enc on y-axis)	94 (slice thickness) on y	6.0 on y
